# Femoral Neck Fractures in Elderly Patients With Coxarthrosis and High Dislocation: The Application of Conservative Treatment

**DOI:** 10.7759/cureus.35805

**Published:** 2023-03-05

**Authors:** Mehmet Akif Akcal, Ibrahim Eke, Tansel Mutlu

**Affiliations:** 1 Orthopaedics and Traumatology, Antalya Atatürk State Hospital, Antalya, TUR; 2 Orthopaedics and Traumatology, Karabük University School of Medicine, Karabük, TUR

**Keywords:** coxarthrosis, the visual analogue scale, harris hip score, developmental dysplasia of the hip, fracture femoral neck

## Abstract

Objective: In this study, our aim was to evaluate the results obtained by conservative treatment of femoral neck fracture in patients with untreated Crowe type 4 coxarthrosis with high dislocation.

Methods: This was was a retrospective study done at the Orthopaedics and Traumatology Clinic in a secondary care public hospital between 2002 and 2022, in Türkiye. Femur neck fractures were evaluated in six patients who had untreated Crowe type 4 coxarthrosis with high dislocation.

Results: In the study, we had six patients with undiagnosed developmental dysplasia of the hip (DDH) who suffered femoral neck fractures. The youngest among these patients was 76 years old. Conservative treatment (bed rest, analgesics, non-steroidal anti-inflammatory drugs, and, if needed, opiates and low molecular weight heparin for antiembolic treatment) was found to reduce Harris Hip Score (HHS) and Visual Analogue Scale (VAS) scores significantly (p<0,05). Stage 1 sacral decubitus ulcer occurred in two (33.3%) patients. Patients acquired daily activity capacity similar to their situations before fracture within five to six months. None of the patients suffered embolisms and there was no union in the fracture line of the patients.

Conclusion: Based on our data, we think that conservative treatment is a remarkable option for these patients, as the complication risks are low and positive results can be obtained. Thus, we may conclude that conservative treatment can be considered in femoral neck fractures of elderly patients with DDH.

## Introduction

Hip fractures are one of the common causes of morbidity and mortality around the world. Fractures of the femoral neck represent a significant portion of these fractures [1]. The incidence of proximal femur fracture increases with age. Most patients with hip fractures are above 80 years of age and about 75% are females. In young adults, these fractures occur as a result of high-energy trauma or underlying pathological causes. In the elderly population, fractures are often associated with low-energy traumas; 90% of hip fractures in the elderly occur after simple falls [2-4]. In young patients, treatment is a closed reduction or internal fixation. In older patients, treatment varies according to patient profile, underlying disease, the choice of the patient and the surgeon [5,6], and health policies of the particular region. Conservative (with or without traction) or surgical treatment can be applied [7]. In some studies, it has been shown that conservative treatment provides good results in high-risk patients [8].

High dislocated femur fractures in the elderly are not common occurrences in developed countries due to effective screening programs [9]. In developing countries, we can still encounter some cases in patients who are located in rural areas. For proximal femoral fractures in congenital hip dislocation, the surgical approach has been applied with unsatisfactory results, especially in geriatric cases in which morbidity risk is high and unexpected outcomes may develop. Also, there is an obvious economic burden related to surgical treatment and post-surgery complications.

In this study, our aim was to evaluate the results obtained by conservative treatment (bed rest, analgesics, non-steroidal anti-inflammatory drugs, and, if needed, opiates) of femur neck fracture in patients with untreated Crowe type 4 developmental dysplasia of the hip (DDH).

## Materials and methods

Study design and participants

This was a retrospective study done at the Orthopaedics and Traumatology Clinic in a secondary care public hospital, Atatürk Public Hospital, in Antalya, Türkiye, between 2002 and 2022. Femur neck fractures were evaluated in six patients who had untreated Crowe type 4 DDH. In their anamnesis, it was informed that all the patients had a sedentary life before the hip fracture due to untreated DDH-induced pain and advanced age. Also, it was determined that all of the patients had high Charlson Comorbidity Index points. The Charlson comorbidity index is the most widely used comorbidity index. It was developed to predict one-year mortality among 604 patients based on comorbidity data obtained from a hospital chart review in a single United States hospital [10]. 

Data collection

The patients were clinically evaluated and radiographic images were obtained before the treatment and at the last follow-up. Clinical evaluation included physical examination and the calculation of the Harris Hip Score (HHS) and Visual Analogue Scale (VAS). In physical examination, the strength and mobilization of the hip were evaluated. Radiographic imaging consisted of anteroposterior and lateral x-ray imaging of the pelvis and hip. From these images, the length of the limb and the discrepancy between the limbs were determined. Computerized tomography was also obtained to evaluate the acetabulum and proximal femur of patients. None of the patients was lost to follow-up.

The HHS is a scale developed by Harris that evaluates pain, function, activity, deformity, and range of motion. Although not originally designed for hip arthroplasty patients, it is widely used for this population. This score was evaluated with a rating scale of 100 points; <70 points are considered bad, 70-79 points fair, 80-89 points good, and 90-100 points excellent [11,12].

Ethical approval

Prior to the study, necessary ethical approval was obtained from the Clinical Research Ethics Committee of the University of Health Sciences, Antalya Training and Research Hospital, Antalya, Türkiye. The study was conducted in accordance with the Declaration of Helsinki.

## Results

The mean age of the patients was 82.5 (76-91) years. According to the Garden classification, two of the patients (33.3%) had type 2, three of the patients (50%) had type 3, and one patient (16.7%) had type 4 femoral neck fracture. It was determined that all of the patients had high Charlson Comorbidity Index points and two patients (with 5 Charlson Comorbidity Index points) had 21%, three patients (with 6 Charlson Comorbidity Index points) had 2%, one patient (with 7 Charlson Comorbidity Index points) had 0% 10-year survival (Table 1).

**Table 1:** Demographic and clinical characteristics of the patients

Variables	Patients
Age (years) (min-max)	82.5 (76-91)
Charlson Comorbidity Index	
5 points, n (%)	2 (33.33)
6 points, n (%)	3 (50)
7 points, n (%)	1 (16.66)
Garden Classification	
Type 2, n (%)	2 (33.33)
Type 3, n (%)	3 (50)
Type 4, n (%)	1 (16.66)

In terms of pre-treatment mobilization grades, two patients (33.3%) were found to be mobile with a single walking stick in-house and four patients (66.7%) were mobile twice a week with a single walking stick for about 100 meters. Since patients can be mobilized in an average of four-five weeks, low molecular weight heparin was used for antiembolic therapy for five weeks. After the fracture, patients were initially mobilized within their beds by their relatives whom we trained, to the degree at which they could tolerate pain. They were mobilized out-of-bed with a walker four weeks after the fracture.

The follow-up duration of the patients was 11.1 (6-16) months. VAS and HHS scores are shown in Table 2. After conservative treatment, HHS increased and VAS values decreased significantly (p<0.05) (Table 2).

**Table 2:** Comparison of visual analogue scale and Harris hip score of the patients after fracture and at final follow-up

Patients	Visual analogue scale	Harris hip score
After Fracture	8.8 (7-9)	30.3 (22-34)
Final Follow-up	2.7 (2-4)	59.6 (52-66)
p-value	<0.05	<0.05

Stage 1 sacral decubitus ulcer occurred in two (33.3%) of the patients. Patients acquired daily activity capacity similar to their situations before fracture within five to six months. None of the patients suffered embolisms and there was no union in the fracture line of the patients.

## Discussion

Although new cases are rarely encountered over the last 10 years due to screening programs for DDH in newborns, it is, unfortunately, seen at an endemically high incidence in Türkiye compared to most European and American countries at older ages. Today, DDH is a significant proportion cause of young adult hip osteoarthritis [13].

In the literature, there is no study on the treatment methods for femur neck fractures in patients who have untreated DDH. The reason is thought to be the fact that early diagnosis and treatment of DDH at all ages has increased today; thus, reaching old age with this disease untreated is a very rare occurrence. In our study, we had six patients with undiagnosed DDH who suffered femoral neck fractures. The youngest among these patients was 76 years old. Conservative treatment was found to reduce VAS values and increase HHS. Furthermore, our patients were able to reach their previous level of daily activity in five to six months.

Another important point is that it is not possible to apply partial prosthesis for such cases and, at the same time, pose significant challenges for total hip replacement. There are various studies in which the risks (frail bone and pathological anatomy) and complications of surgery (nonunion, loosening of prostheses, hip dislocation, and pain) in patients with DDH have been shown [14-16]. In addition, insufficient blood supply of the region due to morphological abnormalities in the femoral head and acetabulum may cause a low chance of union in the surgical treatment of the femoral neck with the internal fixation technique.

Although it is an undisputed issue that early surgical treatment of proximal femur fractures in the elderly without DDH reduces mortality, it has been reported in various studies that surgery-related complications such as wound infection, delirium, non-union, mal-union, wound infection, dislocation, intrusive hardware, nerve palsy, and heterotopic bone formation may develop after surgical treatment [17-19]. And also, based on the technical difficulties derived from high hip dislocation, non-surgical management might be worth considering for older patients with hip fractures in this setting. 

Although the number of patients in our study is very low, we believe our results show that conservative treatment of femoral neck fractures appears to be a reasonable option in elderly patients with high hip dysplasia.

There are studies suggesting the conservative treatment of femur neck fractures in non-DDH hip fractures in elderly patients. In a study by Helbig and colleagues, involving 54 patients with femoral neck fractures, it was reported that 53% of the patients did not develop any complications with conservative treatment, and there was no difference between conservative and operative treatment in terms of survival rate, score, and patient satisfaction [20].

In a study comprising patients older than 65 years, Xu et al. compared surgical and conservative treatment in Garden type 1 and 2 femoral neck fractures. Surgical treatment revealed a higher degree of union; however, regarding the portion of patients who had moderate or severe pain, conservative treatment was found to be better than surgical treatment (51.3% and 61.3%, respectively). They suggest conservative treatment in elderly patients or when there is a risk involved in surgery [21].

Stability of the hip in Crowe type 3 and 4 DDH is obtained by ensuring femoral head and acetabulum compliance in addition to achieving a suitable relationship between the trochanter major and soft tissue. Therefore, when the femoral neck is broken, the physical load is still on the capsule and trochanter major, so stability is not greatly affected.

We also did not prefer surgical treatment because our patients were sedentary, and the femur head and neck were less effective in carrying the body burden. Although there was no union at the fracture line in patients, we found that they reached daily activity similar to their state before the fracture. In addition, a significant decrease in VAS scores was observed in the last follow-up of patients.

The fact that we have only a few patients in our study and that there are no surgically-treated patient groups available for comparison are major limitations of our study.

## Conclusions

Based on our own data, we think that conservative treatment is a remarkable option for elderly patients, as the complication risks are low and positive results can be obtained. Thus, we may conclude that conservative treatment can be considered in the femoral neck fractures of such patients with DDH.

We think that the results of our research will contribute to the literature. However, studies with a larger patient population comparing the conservative treatment and surgical treatment of femoral neck fractures in elderly patients with untreated Crowe type 4 DDH will be instructive for orthopaedic surgeons.
